# Honey bees long-lasting locomotor deficits after exposure to the diamide chlorantraniliprole are accompanied by brain and muscular calcium channels alterations

**DOI:** 10.1038/s41598-019-39193-3

**Published:** 2019-02-15

**Authors:** Aklesso Kadala, Mercédès Charreton, Pierre Charnet, Claude Collet

**Affiliations:** 1 INRA, UR406 Abeilles et Environnement, Toxicologie Environnementale, 84914 Avignon, France; 2 UMT PRADE, Protection des Abeilles dans l’Environnement, 84914 Avignon, France; 3 IBMM UMR CNRS 5247, Université de Montpellier, 34293 Montpellier, France

## Abstract

Diamides belong to one of the newest insecticides class. We characterized cellular effects of the first commercialized diamide, chlorantraniliprole (ChlorAnt). ChlorAnt not only induces a dose-dependent calcium release from internal stores of honey bee muscle cells, but also a dose-dependent blockade of the voltage-gated calcium current involved in muscles and brain excitability. We measured a long lasting impairment in locomotion after exposure to a sublethal dose and despite an apparent remission, bees suffer a critical relapse seven days later. A dose that was sublethal when applied onto the thorax turned out to induce severe mortality when applied on other body parts. Our results may help in filling the gap in the toxicological evaluation of insecticides that has recently been pointed out by international instances due to the lack of suitable tests to measure sublethal toxicity. Intoxication symptoms in bees with ChlorAnt are consistent with a mode of action on intracellular calcium release channels (ryanodine receptors, RyR) and plasma membrane voltage-gated calcium channels (Ca_V_). A better coupling of *in vitro* and behavioral tests may help in more efficiently anticipating the intoxication symptoms.

## Introduction

Ryanodine receptors (RyR) are intracellular calcium release channels that are ubiquitously expressed in tissues of both vertebrates and invertebrates. In mammals, three RyR isoforms are encoded by three different genes (*ryr* 1 to 3), whereas in invertebrates a single *ryr* gene has been identified^1^. RyRs operate under the strict control of cell-specific stimuli and mobilize calcium from intracellular organelles (endoplasmic and sarcoplasmic reticulum) sequestrating this ion at high concentrations (mM levels). At rest, cytoplasmic calcium is maintained in a cell at a low level (close to 100 nM) and RyR-dependent release of calcium from intracellular stores can increase this concentration by 10–1000 fold. Cytoplasmic calcium then induces multiple signaling pathways, including skeletal muscle and cardiac contraction, neurotransmitters and neurohormones release, and gene expression for example. RyRs have a low and a high affinity binding sites for calcium that provide a bell-shaped cytoplasmic calcium dependence of channel opening, with 0.1–1 µM calcium inducing channel opening while 1–10 mM produces closure^2,3^. As in mammalian cardiac muscles, in bee muscle fibers, RyRs opening is mainly controlled by voltage-gated calcium channels by a so called calcium-induced calcium release (CICR) mechanism, where calcium entering through plasma membrane CaVs over the surface membrane and along the T-tubules will activate RyRs^4,5^. In neurons, RyRs are modulated by cADPR (cyclic ADP-ribose). RyR can also be modulated by plant compounds such as ryanodine (formerly used as insecticide but discontinued in the 50’s owing to strong mammalian toxicity), caffeine, xanthines^4,6^. In addition, RyR can be modulated by endogenous effectors and accessory proteins, pH and ionic strength^2^. Several mutations in the human RyR genes are accompanied by pathologies such as malignant hyperthermia and central core disease, where channels susceptibility to physiological stimuli or to environmental stressors (e.g. stress or volatile anesthetics) are increased, and need for rapid pharmacological intervention to avoid death, for instance with dantrolene, a compound that inhibits calcium release from internal stores^7^. Pivotal roles of bee RyRs have been functionally identified in honeybee eye photoreceptors^8,9^, in muscles^4^, in the brain and in the heart^10,11^.

Chlorantraniliprole is the first commercialized member of the anthranilamic diamides class of insecticides. It has been approved for use in the European Union in 2014 and for ten years (EU Pesticide Database and Reg. EC No 1107/2009). The molecule is also described in the literature as Cpd 14, product 1a, D3, DPX-E2Y45 or Rynaxypyr (CAS Number 500008-45-7) and is used in commercial formulations, including Coragen® and Altacor®^12–16^. Several other members of the anthranilamic diamide family have also been described^17,18^. The diamides are widely used (diamides market value ~ 1.4 billion US dollars in 2015 versus ~4.6, 2.8, 1.3 billion dollars for neonicotinoids, pyrethroids and avermectins respectively) and presented as efficient against some pests especially in the context of resistance to other insecticide classes^19–21^. However, the ‘EFSA conclusion on pesticide peer review’ for chlorantraniliprole^22^ underlined the lack of ‘agreed test guidelines for assessing its sublethal effects’ in the Draft Assessment Report (http://dar.efsa.europa.eu/dar-web/provision)^23^. The molecular mode of action of diamides (anthranilic and phtalic diamides) was concurrently identified by several agrochemical firms^24^ and a new category of insecticide mode of action (MoA group 28) was created by the Insecticide Resistance Action committee in their classification scheme (IRAC, a group including most of crop protection industries). Diamides act as RyRs agonists and induce release of calcium from intracellular stores. The rise of anthranilamides and other compounds targeting RyR was boosted by the 2004 patent application WO2004027042 that described the expression of a number of insect RyR full length genes in insect cell lines^14,15^. Most of these genes are those from the so called ‘insect pests’. A major breakthrough was achieved in 2000 with the successful (although transient) Drosophila RyR gene expression leading to production of a full length or partial (C-term) RyR in Chinese hamster ovary cells^25^. More recently, full-length RyRs from insects were afterward cloned, namely *PxRyR, CmRyR, OfRyR, BdRyR SfRyR*, *LdRyR*, and *SeRyR*^26,27^. However, to our knowledge, the bee RyR has never been neither cloned nor expressed in insect cell lines and the functional effects of chlorantraniliprole on RyRs from useful insects such as bees not extensively tested. Indeed, calcium release assays were made either on neurons from cockroach or on insect cells lines expressing RyR from the so called pests, but not on cells from beneficial insects (e.g. pollinators) such as bees. Early symptoms of honeybee intoxication with chlorantraniliprole include death, lethargy, apathy, slow and uncoordinated movements^23^. However, mandatory toxicology tests remain so far rather descriptive when dealing with behavioural effects and preclude from an accurate quantification of sublethal deleterious effects.

Our study gives insights into the molecular mode of action of chlorantraniliprole in bee neurons and muscle cells. Quantitative locomotor approaches shed a new light on the deleterious sublethal effects of this molecule, the first commercialized anthranilic diamide insecticide.

## Results

### Molecular mode of action of chlorantraniliprole in honey bee neurons and muscle cells

**Figure 1:**
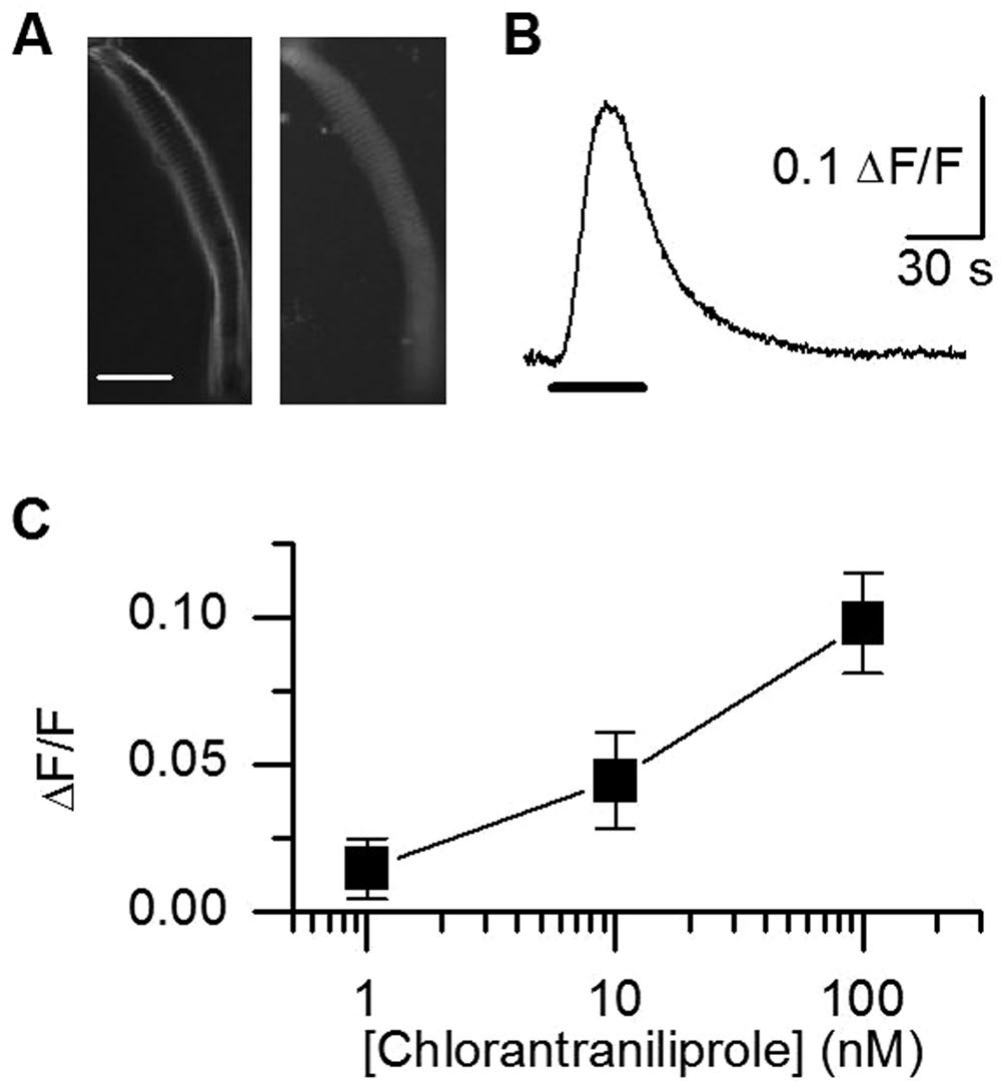
Calcium transients induced by chlorantraniliprole in legs muscle cells. (**A**) Fluo-3 loaded muscle cell seen in transmitted white light (left) and with fluorescence (right). Scale bar: 40 µm. (**B**) Calcium transient induced in a muscle cell upon perfusion with chlorantraniliprole 100 nM. (**C**) Maximal amplitude of calcium transients induced upon perfusion with increasing concentrations of chlorantraniliprole (mean ± SEM, n = 4, 4 and 11 cells for 1, 10 and 100 nM respectively).

To characterize the potency of chlorantraniliprole in inducing calcium release through honey bee muscle ryanodine receptors, muscle fibers isolated from the bee tibia were loaded with the intracellular calcium indicator Fluo-3 (Fig. 1A). Muscle cells were perfused with increasing concentrations of chlorantraniliprole (1 nM, 10 nM, 100 nM, 30 seconds) and calcium was measured as a ΔF/F ratio (Fig. 1B). On average (Fig. 1C), chlorantraniliprole 100 nM induced transients reaching a peak value of 0.098 ± 0.017 ΔF/F (n = 11 cells, 0.03–0.18). At the time scale of our experiments, only the concentration 100 nM could induce contracture upon long-lasting perfusion (3/11 cells). On the contrary, upon two consecutive applications of 100 nM (with a delay of 30 seconds), the second calcium transient was smaller (5/11 cells), suggesting depletion of the sarcoplasmic reticulum along with multiple challenges with this molecule. This depletion or desensitization is evidenced by a transient increase in [Ca^2+^]_i_ and a return to baseline along long-lasting application (80 s). Chlorantraniliprole effect was dose-dependent as 10 nM induced weaker transient increases and 1 nM even weaker ones (n = 4 cells in both conditions). We did not attempt at measuring calcium transients for concentrations higher than 100 nM because these concentrations would induce contraction of the cells (preventing accurate measurements of Ca^2+^-dependent fluorescence variations), but this concentrations range is consistent with available EC_50_ obtained in the literature in non-contracting cell lines expressing an insect ryanodine receptor. In a second set of experiments (muscle cells not loaded with Fluo-3), chlorantraniliprole was found to induce cell contracture at higher concentrations. In the experimental petri dishes, the perfusion tip was approached close to an intact resting cell well attached to the bottom of the dish and the cell was observed through the eyepiece of the microscope while the perfusion flow was turned from control solution (Tyrode without calcium) to 10 µM chlorantraniliprole. The control solution alone was not able to induce any contraction (n = 227, not illustrated). From eight experimental dishes, when the very first cell was challenged with the insecticide, 75% of muscle fibers (6 out of 8) showed strong contractions to contractures upon perfusion. In each dish, a second cell was systematically challenged with chlorantraniliprole (chosen far away from the first one) and only 3 out of 8 cells responded clearly, suggesting a possible desensitization due to diffusion of chlorantraniliprole into the dish. To sum up, if one considers the 2 first cells of each dish, 9 out of 16 cells responded to chlorantraniliprole 10 µM (56%). In another experiment, caffeine, a RyR activator, induced contracture in 56% fibers (n = 129) at a concentration of 5 mM and 77% of cells at a concentration of 20 mM (n = 26, not illustrated). Fibers that did not contract in response to chlorantraniliprole, did not respond to caffeine either, suggesting a similar mode of action for these two compounds (n = 18, not illustrated).

**Figure 2:**
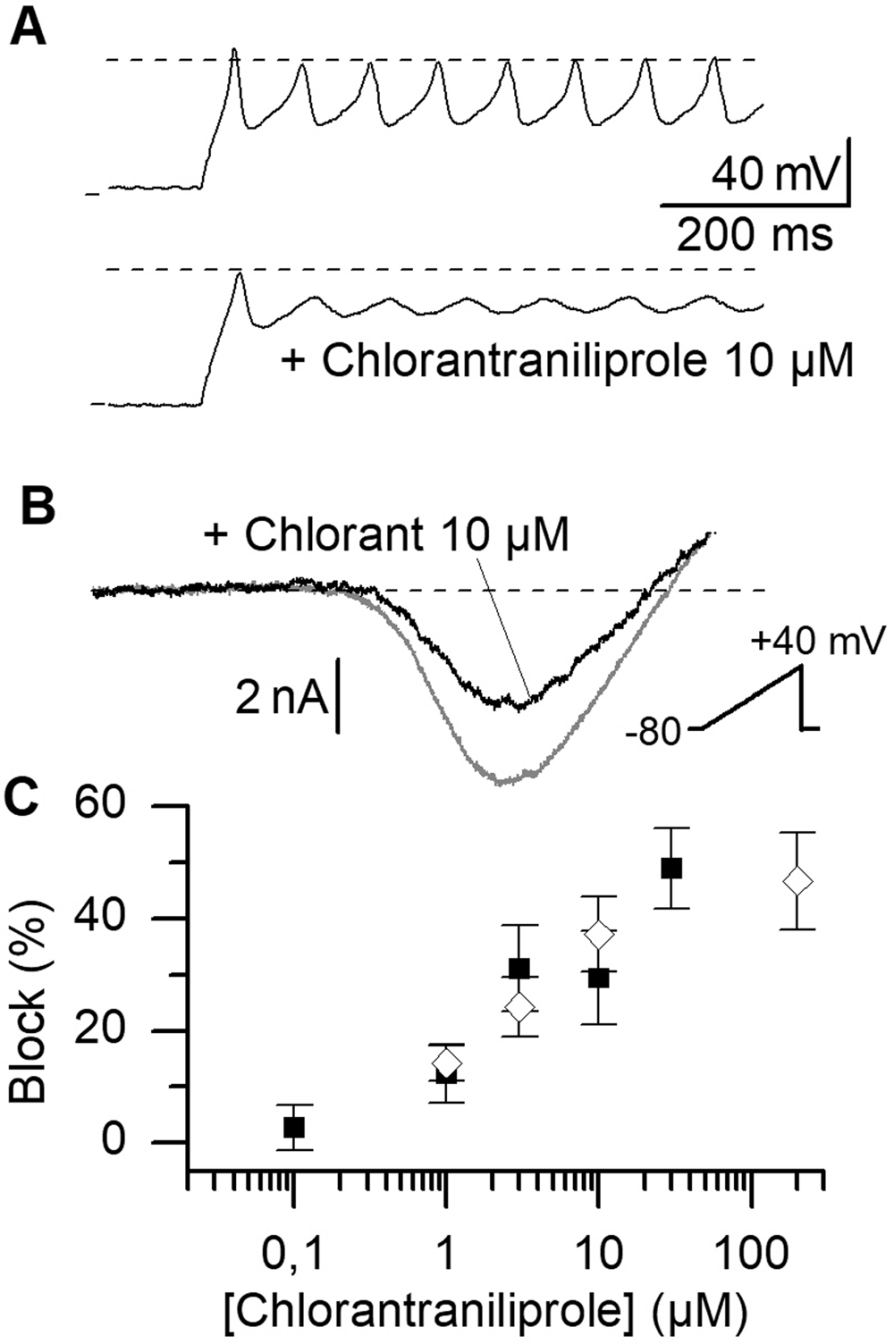
Action potentials and high-voltage-activated calcium channels are blocked by chlorantraniliprole. (**A**) Calcium action potentials in current-clamp (top trace) are decreased in amplitude after exposure to chlorantraniliprole 10 µM in muscle cells. (**B**) The amplitude of the current through voltage-gated calcium channels (CaVs) is decreased by chlorantraniliprole (voltage ramps obtained under voltage-clamp in a muscle cell). (**C**) Dose-dependent blockade of CaVs (mean ± SEM, n = 6–13 per concentration) in neurons (open diamond) and muscle cells (black squares). DMSO concentration was 0.1% in all conditions except at 30 and 200 µM, were DMSO was increased to 1.5 and 5%, respectively, to overcome the low water solubility of the insecticide.

Although diamides are repeatedly described as specific ryanodine receptor activators (since their first description in 2006), few experiments have actually been performed to challenge this hypothesis^17^. In bee muscle fibers, excitation-contraction coupling relies primarily on the activation of voltage-gated calcium channels producing the primary increase in cytoplasmic calcium responsible for RyR opening through a CICR mechanism^4^. The fact that contraction was impaired in bees exposed to chlorantraniliprole^23,28^ may thus also rely on a modification of the calcium entry through CaVs. We explored this possibility by recording CaV activity using the patch-clamp method on isolated muscle fibers. In current-clamp experiments, perfusion with 10 µM chlorantraniliprole decreased the amplitude of action potentials recorded in a Tyrode solution (Fig. 2A, with 10 mM EGTA in the patch pipette to prevent cell contracture). In voltage-clamp we used appropriate extra and intracellular solutions designed to block all Na, Cl and K conductance and barium ion as permeant ion through CaVs to record activity in muscle cells in the presence of increasing concentrations of chlorantraniliprole (0.1 to 10 µM). In these conditions, CaV activity could be recorded in muscle cells during more than 10 minutes without any sign of significant current run-down. Perfusion with chlorantraniliprole induced a dose-dependent block of the current. Chlorantraniliprole 10 µM blocked 29 ± 8% of the current (n = 6, Fig. 2C, black squares), consistently with its effect on action potentials. A similar effect was also obtained in neurons, with 37 ± 7% (n = 7) blockade of the current in central neurons at 10 µM (Fig. 2C, open diamonds). At higher concentrations (also tested in previous studies^12^, and necessitating DMSO concentrations >0.1% to avoid problems with the weak solubility of chlorantraniliprole in water), 30 µM chlorantraniliprole blocked 49 ± 7% of the muscle current (n = 4, with 1.5% DMSO). In neurons, chlorantraniliprole 200 µM blocked 47 ± 9% of the current (n = 7, with 5% DMSO).

### Long-lasting toxicity following an acute exposure to chlorantraniliprole

**Figure 3:**
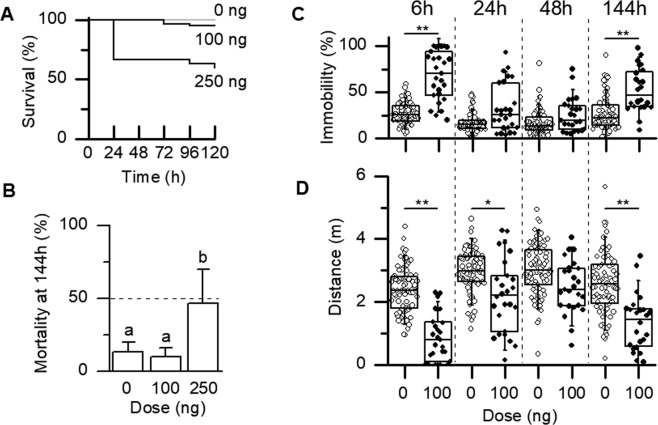
Classical toxicology of chlorantraniliprole and locomotor deficits induced at a sublethal dose. (**A**) Bees exposed according to current mandatory classical mortality tests experienced a severe decrease in survival for doses >100 ng/bee during a long-lasting period of observation (6 days). In this survival test, all control bees (grey line) were alive after 6 days (120 h). (**B**) Seven days after exposure, the dose 100 ng induced a level of mortality not more elevated than the control exposure and is thus retained as a sublethal dose at 144 h (SLD_144h_), whereas 250 ng was highly toxic (p < 0.01, mean ± SEM). Different letters indicate a significant difference. (**C**) A daily locomotor test revealed that a dose characterized as sublethal at 7 days (SLD_144h_) increased periods of prostration 6 hours and 7 days after exposure (p < 0.001) with a transient attenuation of symptoms at day 2 and day 3. In box and whiskers graphs, individual data are presented as circles (open and filled for control and exposed bees, respectively), boxes show quantiles and whiskers the minimum and the maximum values. (**D**) Periods of prostration were not compensated by periods of intense phases of activity, since the distances covered by bees during active walking was decreased, not only at 6 h and 7 days after exposure, but at 24 h as well (p < 0.001, p < 0.05 and p < 0.001 respectively). An apparent but transient recovery was thus observed at day 2 (48 h).

Classical toxicological laboratory tests with caged bees kept in an incubator revealed that doses ≥250 ng/bee trigger high levels of mortality (Fig. 3A), in contrast with formerly published literature^23^. At 48 h after exposure, the dose 250 ng/bee induced a significantly higher mortality (33 ± 12%) than in control (p < 0.001, exact Fisher test, 0 dead bees out of 60 in control). A 2.5-fold weaker dose (100 ng/bee) did not induce more mortality than in control conditions up to 7 days after exposure i.e. at 144 h (Fig. 3B, p = 1.000, exact Fisher test, 4/30 and 3/30 dead in the control and exposed group respectively). This dose was thus retained as a sublethal dose over a period of 7 days after exposure (SLD_144h_) to explore possible long-lasting symptoms of intoxication after an acute exposure to this insecticide.

### Quantification of intoxication symptoms at a sublethal dose in a locomotor assay

Immediately after exposure to the selected chlorantraniliprole sublethal dose SLD_144h_, some bees showed signs of locomotor deficits (never observed in the control group), with prostration and slow movements. The intoxication symptoms happen to last for several days in a fraction of bees. In order to actually and objectively quantify these deficits, we used two laboratory assays: i) a locomotor test and ii) a reflex-contraction test (see below). For the locomotor test, each bee was introduced in a vertically disposed Petri dish placed in the dark, with a light coming from above and videos were recorded during 3 minutes. Each individual locomotor behavior was observed in the long run after exposure, i.e. at 6 h, 24 h, 48 h and 144 h. The percentage of time spent in a stationary position, was measured as a proxy of prostration (see Methods for a definition of the immobility threshold). Experiments were performed daily for 6 days on a total of 27 bees exposed to the SLD_144h_ and compared to experiments performed on control bees. Six hours after exposure to a SLD_144h_ (100 ng/bee), immobility was very frequent (Fig. 3C, 68 ± 5% of observation time, n = 27 bees), more than twice higher than in control conditions (27 ± 5%, n = 81 bees, p < 0.001, Kruskall-Wallis and Dunn’s multiple comparisons test). This effect was transiently attenuated at day 2 and 3 after exposure, but a statistically significant relapse was observed at day 7 (144 h, p < 0.001), indicating a long-lasting intoxication effect after a single exposure.

To go further into the characterization of locomotor deficits, the total distance covered was measured for each bee, excluding distances somehow performed at a speed under the threshold of immobility to get the performances of bees during actual walking, excluding periods of immobility, crawling or slow wandering (Fig. 3D). The total distance covered was indeed on average decreased by 63%, 25% and 48% at 6 h, 24 h and 6 days after exposure, respectively (p < 0.001, p < 0.05, p < 0.001). A slight but not significant decrease was also observed at 48 h (a 18% decrease). These results show that during a long period of observation between 6 h and 6 days after exposure, bees are not able to compensate from their increased periods of immobility (Fig. 3C) by improving performances during active walking phases.

### Quantification of intoxication symptoms at a sublethal dose with a reflex-contraction test

**Figure 4:**
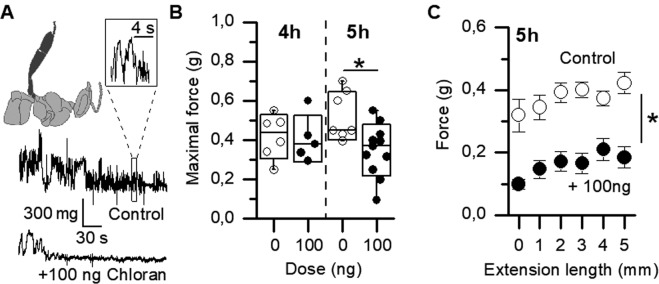
Impairment in leg contraction several hours after exposure to a sublethal dose. (**A**) Measurements of leg muscles contraction. Only the left metathoracic leg of the bee is shown, for clarity. Maximal force was measured on a series of stretching length from 4 to 10 mm at 4 and 5 h after exposure. In control, contractions are fast and powerful (top trace, 5 h), whereas 100 ng chlorantraniliprole decreases force production (bottom trace). (**B**) Maximal force production is significantly decreased at 5 h after exposure to a 100 ng dose, but not at 4 h (same box plot characteristics as in Fig. 3). (**C**) Five hours after exposure to a 100 ng dose, force production is consistently decreased at each stretching length (mean ± S.E.M., n = 7 and 11 for control and 100 ng/bee, respectively).

In order to explore the mechanism by which a locomotor deficit takes place, we measured directly the force produced by a reflex muscular contraction of the third pair of leg after stimulation of a harnessed bee (see Methods and Fig. 4A). Force was measured at several extension lengths and the maximal force was kept for each bee. Four hours after exposure to the SLD_144h_, no significant effect was observed (Fig. 4B), whereas at 5 hours after exposure, a significant decrease (39%) was evidenced by the test, with an average maximal force of 0.53 ± 0.05 and grams and 0.35 ± 0.04 grams in control and exposed groups, respectively (p < 0.01, Mann-Whitney, n = 7 and 11). The maximal force was not necessarily obtained at the maximal extension length. Average force at each extension length was ~2-fold weaker after exposure to chlorantraniliprole (Fig. 4C).

### Exposure route influences toxicity of chlorantraniliprole

Finally, since both RyR and CaV functions are altered in muscle cells and neurons, one could expect that any organs expressing those channels could be altered as well. A mortality test for chlorantraniliprole was thus tested by application to eyes, antenna and abdomen and compared to the classical exposure *via* the thorax. Interestingly, the dose retained as a ‘thorax SLD144h’ (sublethal at 144 h when applied on the thorax) turned out to induce some mortality at 24 h when applied to the other body parts, with an increasing lethality for thorax < eye < antenna < abdomen (0, 4, 11 and 13 dead bees out of 30 in each group respectively). The group in which chlorantraniliprole was applied on the thorax showed no more mortality than its control group, whereas the effect was significant in the abdomen and antenna groups, (Fisher exact test, p < 0.001), but not in the eye group.

## Discussion

We provided the first characterization of bee RyR functional sensitivity to chlorantraniliprole by measuring the calcium release in muscle cells isolated from the leg. According to our results, the muscle RyR can be activated by concentration as low as 1 nM, and a dose-dependent activation up to 100 nM has been measured. These results are consistent with chlorantraniliprole EC_50_ values in the tenth of nanomolar range in cockroach neurons and insect cell lines expressing heterologous insect RyRs such as the fly *Drosophila melanogaster* or the moth *Heliothis virescens*^12,22,29^. At higher concentrations, muscle cell contracture precluded from accurate calcium release measurements. The RyR agonist caffeine at 20 mM and chlorantraniliprole at 10 µM both induced contraction in ¾ of bee muscle cells, suggesting that the synthetic insecticide is 2000-fold more potent on muscle than the natural plant alkaloid. Under voltage-clamp conditions, we have shown earlier that the natural plant alkaloid ryanodine (5 µM) was interfering with normal calcium release in the absence of any effect on the voltage-gated calcium current^4^, whereas exposure to chlorantraniliprole in the micromolar range (3–10 µM) induces a depression in the calcium current amplitude and in action potentials as well (Fig. 2). Although the site of action of ryanodine and chlorantraniliprole have been both identified on the RyR channel, their functional effect is thus quite different and binding experiments conducted with bee, fly and moth RyR suggest interspecific binding differences^30^. Our study provides original insight into insect muscle cell sensitivity, since muscle cells sensitivity to chlorantraniliprole was so far mainly extrapolated from a mammalian muscle cell line (C2C12 myotubes). It would be interesting to test the effect of this insecticide class on freshly isolated cardiac or skeletal muscle fibers from mammalians. Exposure of neurons and muscle cells to chlorantraniliprole is accompanied by voltage-gated calcium channels blockade. Whether this is a direct effect or a consequence of effects on RyRs remains to be elucidated. In response to concerns raised in the EFSA publication entitled ‘conclusion on the peer review of the pesticide risk assessment of the active substance chlorantraniliprole’^22^ about effects of chlorantraniliprole at low doses, we provide new sublethal tests that bring new toxicological results on diamides. With a contraction test and a locomotion test, we have identified early symptoms at the sublethal dose 100 ng/bee, as well as long-lasting effects, including a critical relapse 7 days after exposure. Our classical mortality test suggests stronger contact toxicity than reported earlier for bees exposed to chlorantraniliprole. Indeed, in former studies^23,28^, the LD_50_ was not reached at the highest dose tested (34% mortality at 120 h with 4000 ng/bee applied on the thorax), and doses below 500 ng/bee produced almost no mortality (0–2% at 120 h, acetone as a solvent). Tentative adjustment of a logistic model on available EFSA data^22^ and assuming that all individuals in the population are susceptible would lead to an estimation of LD_50_ around 8 µg/bee. In our conditions, the dose 250 ng/bee produced a strong toxicity (33 ± 12%), suggesting a leftward shift of the dose-mortality relationship of ~16 fold in our case. Moreover, the sublethal dose that we retained in our study for thoracic exposure (SLD_144h_) turned out to produce mortality (up to 43%) when applied on other organs rich in muscles and neurons most likely expressing ryanodine receptors and voltage-gated calcium channels (antenna and abdomen). This result suggests that the exposure zone recommended in OECD mandatory tests (*i.e*. thorax) should be reconsidered in the case of diamide insecticides. Finally, chlorantraniliprole produces a number of metabolites^22^ that could be tested with our methods. Since calcium ions coming from RyR and CaV routes are involved in several processes including tissular maturation or hormone secretion, a possible explanation in the long lasting effects observed after a single exposure could be found in structural defects in the nervous system, in muscles, the heart or even endocrine disruption.

## Conclusion

Insecticides targeting calcium release channels are now widely used and many other prototypes have been proposed on the basis of *in vitro* screening on heterologously expressed receptors. Our present description of sublethal effects on useful insects underline the necessity of extensive *in vivo* analysis prior to any release of these molecules in the environment. Screening should thus be performed both on *in situ* and on heterologously expressed receptors. The fact that now, many of *Apis mellifera* channels and receptors can be expressed (CaV1 to 3, NaV1, CaV4-DSC1)^31–37^ provides a very useful library of excitable models to complete these *in vivo* toxicological analysis by the construction of a sensitive-channels pattern helpful to fully understand the functional effects of these molecules.

## Material and Methods

### Honey bees

For classical toxicological tests (mortality), sublethal tests, and for imaging and electrophysiology assays on muscle cells, newborn honey bees (*Apis mellifera*) were collected during the summer season and pooled from two hives (#115 and 56) maintained in the experimental apiary of the *Abeilles & Environnement* Research Department on the Avignon INRA PACA campus. To collect newborn bees, four frames of developing brood were gently brushed to get rid of adult bees and placed into an incubator overnight (30 °C, high humidity) in order to harvest newly emerged bees the next morning. For electrophysiology experiments on neurons, brains came from bees at the pupal stages P4-P6, collected with entomological forceps directly in the apiary, from brood combs of hive #115. Colonies received a treatment against *Varroa* (Apivar^TM^, active ingredient amitraze) and were healthy, without any obvious symptoms of disease.

### Acute exposure of bees to insecticides

Technical-grade chlorantraniliprole (the active ingredient, CAS number: 500008-45-7) was purchased from Sigma (analytical standard, purity >98%). Chlorantraniliprole was dissolved in acetone and briefly sonicated at a concentration of 3 µg/µl (below its maximal solubility, according to literature), and final concentrations were obtained by successive acetone dilutions in amber glass vials thoroughly vortexed at each step. Solutions were stored at −20 °C. Exposure to chlorantraniliprole was performed between 8 and 10 am. Newborn bees pooled from four frames (as described above) were anaesthetized with CO_2_ (batches of bees were exposed to a controlled volume of CO_2_ for 30 seconds in an anesthesia induction chamber). They were placed on ice in order to maintain lethargy and 1 µl solution (pure acetone containing either 0, 100 or 250 ng/µl chlorantraniliprole) was applied to the dorsal part of the thorax with a Hamilton syringe mounted in a repeating dispenser. Full acetone evaporation was fast and bees were placed in standard plastic cages 10.5 cm × 7.5 cm × 11.5 cm, modified from^38^ in a ventilated incubator (29 °C, 40% humidity, dark) and provided with *ad libitum* water and sugar paste (Apifonda, Ickowicz – sucrose 85%, glucose 5%, fructose 3%, water) saturated with powdered sugar (Daddy Cristalco) to lower viscosity. Mortality tests were performed prior to the reflex-contraction and the locomotion assays in order to determine a sublethal dose (SLD) close to the onset of the dose-mortality relationship. Two replicates of 30 bees were used at each application doses (i.e. twice the number of bees required in marketing authorization application –MAA- mandatory tests). The sublethal range was defined as doses producing a mortality level not statistically different from the control 144 hours (7 days) after exposure, while a dose 2.5-fold higher than the SLD_144h_ caused mortality significantly higher than the control 144 hours after exposure. In one test, the toxicity of this SLD (when applied on the thorax) was explored on other areas of the body: antennae, eyes or abdomen.

### Video-tracking analysis

Locomotor activity was quantified from bees at day 1, 2, 3 and 7 (6, 24, 48 and 144 h) following exposure to chlorantraniliprole (dose SLD144h) or acetone alone. For each observation day, bees were monitored for 3 minutes using a webcam controlled with VirtualDub (GNU free software, acquisition frequency 1 Hz). The arena set up allows the simultaneous video-tracking of nine bees, every 5 minutes (3 minutes of effective video tracking and 2 additional minutes to transfer the bee from their cage to the arenas, to allow for short time acclimation, and to transfer them back to their cage and incubator at the end of the tracking). Video tracking was performed in the afternoon and at each day of observation and three replicates of nine bees exposed to the SLD_144h_ were submitted to the test. The vertical individual observation arenas, consisted in Petri dishes (diameter 10 cm), aligned on 3 columns on a white board. A dark chamber was placed around the device in order to avoid any behavioural variation due to daylight from outside or artificial room light intensity. Arenas were illuminated from above (two parallel flicker free LED ramps, length 10 inches, 9 LED each, for a total of 0.72 W, 70 lumens of cold light, StarLED sticks, Starlicht, Germany). Experiments were done at room temperature (22–24 °C). Videos were semi-automatically analyzed using ImageJ (open source, Rasband WS, National Institutes of Health, Bethesda, USA) with a custom made script using available filters and plugins in order to obtain a series of x,y coordinates for each bee. Individual paths were afterwards processed with Excel.

### Reflex contraction test

Bees were individually cold-anaesthetized and harnessed in a commercial tube cage, an end of which is closed with a soft mesh. A soft plastic foam-covered plunger allowed gently squeezing their body, when lying on their back. One of the metathoracic legs was passed through one slightly enlarged mesh of the net to allow for free movement of the leg only. The tarsa was clamped with a small vessel clip (WPI) and attached to a force transducer (Grass FT03) with a thread. The force transducer was connected to a digitizer (BIOPAC systems, France) and experimental recordings were acquired at 400 Hz and analyzed with the software AcqKnowledge (BIOPAC systems, France). A force calibration performed according to the recommendations of the transducer manufacturer allowed to convert mV into grams. Maximal force production was systematically recorded at 6 leg tension lengths, starting from a maximal tension of 5 mm (leg fully extended) down to 0 mm. A reflex contraction was induced by touching the mandibles. Force was measured at 4 and 5 hours after exposure to the SLD_144h_ chlorantraniliprole dose.

### Calcium imaging in muscle cells

Muscle cells were obtained from newborn bees following a procedure described earlier^5^. In brief, muscle cells were isolated from the third pair of legs (metathoracic legs). Bees were first cold-anaesthetized (4 °C, 20 min) and decapitated. Tibias were dissected out in Tyrode (without calcium), the cuticle was cut-opened along the thin edge of the pollen basket and pinned open with minute pins. Muscles were exposed during 20 minutes (37 °C) to a solution containing enzymes (collagenase, papain, pronase, trypsin) and triturated in Petri dishes. Muscle cells were loaded with the membrane permeant acetoxymetylester form of the intracellular Ca^2+^ indicator fluo-3 (Molecular Probes, Invitrogen Corp) as previously described^4^. Loading was performed at 20 °C for 40 min in Tyrode with 5 µM fluo-3 AM (with a 0.1% final DMSO concentration) and extracellular solution was washed out with Tyrode. The muscle cells were placed on the stage of an inverted microscope (DM IRB, Leica). The beam of light from a 100 W mercury bulb was passed through a 450–490 nm bandpass filter and focused on the preparation, providing the excitation light for fluo-3. The fluorescence light was collected by a x20 objective through the use of a dichroic mirror and a longpass filter (>515 nm) and detected with a camera (Qicam CCD, 12-bits, Qimaging Corp, Surrey, BC, Canada). Acquisition of series of images was performed using the Qcapture Pro software (Qimaging Corp, Surrey, BC, Canada) at a frequency of 2 Hz. Images were afterwards processed with the software Image/J (NIH, USA). Background fluorescence was measured from an area distant from the studied cell and subtracted from the corresponding *F*. Fluorescence values were expressed as Δ*F*/*F*_*0*_, *F*_*0*_ being the resting (or baseline) fluorescence and Δ*F* the change in fluorescence from baseline). A chlorantraniliprole stock solution (10 mM) was prepared in dimethylsulfoxide (DMSO). Serial dilutions in a Tyrode solution (Ca^2+^ 2 mM) led to chlorantraniliprole concentrations of 0 (control), 1, 10 and 100 nanomolar (nM) with a final DMSO concentration adjusted to 0.1%. Changes in Δ*F*/*F* were measured in response to solutions perfused onto isolated cells by means of a perfusion system made of 4 polyethylene tubing gathered in a perfusion manifold. In another set of experiments, in which cells were not loaded with the calcium indicator, a higher concentration of chlorantraniliprole (10 µM) was perfused onto cells and the ability of this concentration to induce cell contraction was checked through the eyepiece of the microscope. Similar observations where performed with the control solution and a solution of the RyR-modulator caffeine. In all cases, the DMSO concentration was 0.1%.

### Electrophysiology in neurons and muscle cells

Neurons were obtained from bees following procedures described earlier^39^. In brief, neurons of the antennal lobes were dissected from honeybee pupae brains in a magnesium and calcium-free Tyrode solution and plated on poly-L-lysine coated Petri dished and kept *in vitro* for 2–4 days in a Leibowitz-based cell culture medium (see Solutions). Amplitudes of currents through CaVs were measured with a patch pipette in the whole-cell configuration and an extracellular solution containing barium and blockers of potassium, sodium and chloride channels. Under voltage-clamp, cells were submitted to protocols of 400 ms voltage ramps from −80 to +40 mV. A chlorantraniliprole stock solution (2 mM) was prepared in dimethylsulfoxide (DMSO). Serial dilutions in the extracellular solution led to chlorantraniliprole concentrations of 0 (control), 1, 3 and 10 micromolar (µM) with a final DMSO concentration <0.5%. Changes in the current amplitude were measured in response to solutions perfused onto isolated cells by means of a perfusion system made of 4 polyethylene tubing gathered in a perfusion manifold (maximal changes were assessed at 2 min after the onset of perfusion for each concentration). The current-clamp configuration was used in a set of experiments on muscle cells to record calcium action potentials in control conditions and in the presence of chlorantraniliprole, in a Tyrode 2Ca solution.

### Solutions for cell culture and electrophysiology

The Ca^2+^ and Mg^2+^ -free Tyrode used for neuronal dissection contained (in mM): 140 NaCl, 5 KCl, 10 HEPES, 90 sucrose (pH 7.2). Culture medium was made of a commercial liquid L15 medium (with L-glutamine) supplemented with 5.5 mM D-Glucose, 3.3 mM L-proline, 75 mM sucrose, 10% fetal bovine serum, 1% penicillin/streptomycin (pH 7.2). To record current through CaVs in neurons, the extracellular solution contained (in mM): 105 NaCl, 40 BaCl_2_, 2 MgCl2, 20 TEA-OH, 0.001 Tetrodotoxin, 1 4-amino-pyridine, 10 HEPES, 90 sucrose (pH 7.2). The pipette solution contained 135 CsCl, 5 NaCl, 1 MgCl_2_, 1 CaCl_2_, 10 EGTA, 10 HEPES (pH 7.2). The standard extracellular solution used for patch-clamp in order to isolate the muscular calcium current contained (in mM): 120 TEA-MeSO_3_, 2 BaCl_2_, 2 MgCl_2_, 1 4-aminopyridine, 10 HEPES (pH 7.2). Intracellular (pipette) solution contained (in mM): 140 K-gluconate, 2 MgCl_2_, 10 EGTA, 10 HEPES (pH 7.2). Ca^2+^-free Tyrode solution contained (in mM): NaCl 140, KCl 5, MgCl_2_ 2, HEPES 10 (pH 7.2). CaCl_2_ 2 mM was added to Tyrode to obtain the Tyrode 2Ca. DMSO was added in all perfusion solutions to overcome the poor water solubility of chlorantraniliprole (routinely 0.1% DMSO, for concentrations <10 µM, as stated in Results).

All data generated or analyzed during this study are included in this published article. Data are given as mean ± S.E.M. Mann-Whitney tests were used to compare locomotion data. Exact Fisher tests were used to analyze mortality data.
